# Customized Human Milk Fortification Based on Measured Human Milk Composition to Improve the Quality of Growth in Very Preterm Infants: A Mixed-Cohort Study Protocol

**DOI:** 10.3390/ijerph18020823

**Published:** 2021-01-19

**Authors:** Manuela Cardoso, Daniel Virella, Israel Macedo, Diana Silva, Luís Pereira-da-Silva

**Affiliations:** 1 Nutrition Unit, Maternidade Dr. Alfredo da Costa, Centro Hospitalar Universitário de Lisboa Central, 2890-495 Lisbon, Portugal; maria.cardoso5@chlc.min-saude.pt; 2 Research Unit, Centro Hospitalar Universitário de Lisboa Central, 1169-045 Lisbon, Portugal; danielvirella@chlc.min-saude.pt; 3 Neonatal Intensive Care Unit, Hospital Dona Estefânia, Centro Hospitalar Universitário de Lisboa Central, 1169-045 Lisbon, Portugal; 4 Neonatal Intensive Care Unit, Maternidade Dr. Alfredo da Costa, Centro Hospitalar Universitário de Lisboa Central, 2890-495 Lisbon, Portugal; israel.macedo@chlc.min-saude.pt; 5 Faculdade de Ciências de Nutrição e da Alimentação, Universidade do Porto, 4150-180 Porto, Portugal; diana.mv.silva@gmail.com; 6 Nutrition Lab., Hospital Dona Estefânia, Centro Hospitalar Universitário de Lisboa Central, 1169-045 Lisbon, Portugal; 7 Comprehensive Health Research Centre (CHRC), Medicine of Woman, Childhood and Adolescence, NOVA Medical School, Universidade Nova de Lisboa, 1349-008 Lisbon, Portugal

**Keywords:** body composition, growth, human milk fortification, preterm infants, standard fortification, target fortification

## Abstract

Adequate nutrition of very preterm infants comprises fortification of human milk (HM), which helps to improve their nutrition and health. Standard HM fortification involves a fixed dose of a multi-nutrient HM fortifier, regardless of the composition of HM. This fortification method requires regular measurements of HM composition and has been suggested to be a more accurate fortification method. This observational study protocol is designed to assess whether the target HM fortification method (contemporary cohort) improves the energy and macronutrient intakes and the quality of growth of very preterm infants, compared with the previously used standard HM fortification (historical cohorts). In the contemporary cohort, a HM multi-nutrient fortifier and modular supplements of protein and fat are used for HM fortification, and the enteral nutrition recommendations of the European Society for Paediatric Gastroenterology Hepatology and Nutrition for preterm infants will be considered. For both cohorts, the composition of HM is assessed using the Miris Human Milk analyzer (Uppsala, Sweden). The quality of growth will be assessed by in-hospital weight, length, and head circumference growth velocities and a single measurement of adiposity (fat mass percentage and fat mass index) performed just after discharge, using the air displacement plethysmography method (Pea Pod, Cosmed, Italy). ClinicalTrials.gov registration number: NCT04400396.

## 1. Introduction

### 1.1. Biological Advantages of Human Milk

Human milk (HM) is the first choice for feeding preterm infants [1], as it is the better option that compensates for the immature immune [2,3], vascular [4], and neurological [5] systems. In very and extremely preterm infants, HM has beneficial effects on the neurodevelopmental outcome [6] and preventive effects on morbidities, including retinopathy of prematurity [7], bronchopulmonary dysplasia [8], necrotizing enterocolitis [7,9], and late sepsis [9].

From a nutritional point of view, HM does not fulfill the needs of growing preterm infants, resulting in accumulated nutrient deficits, which may lead to extrauterine growth restriction and poor neurodevelopmental outcomes [10,11]. This is particularly true for protein deficits [12]. Early postnatal growth restriction, in turn, predisposes individuals to late metabolic diseases [11]. To prevent nutritional insufficiencies related to HM while taking advantage of its biological properties, HM multi-nutrient fortifiers are used for preterm infants [13,14,15].

### 1.2. Methods for Fortification of Human Milk

Human milk is fortified using the standard method, which consists of the addition of a fixed dose of a HM multi-nutrient fortifier, according to the manufacturer’s instructions [14]. This method does not take into account the great variability of the nutritional composition of HM [13,14,15,16] and so does not avoid the risks associated with energy–protein malnutrition, poor neurodevelopmental outcome, and metabolic bone disease [17,18,19]. In an attempt to overcome this problem, alternative individualized HM fortification methods have been proposed, particularly target fortification [15].

The target fortification method is based on the regular measurements of the energy and macronutrient contents of HM, in order to customize the target energy and macronutrients to each infant [20]. In addition to the multi-nutrient fortifier, modular protein, carbohydrate, and fat supplements may be added to HM to achieve the desirable nutrient targets for preterm infants [15,21,22].

### 1.3. Questions to Be Answered

Many authors [21,22,23,24] consider the target HM fortification to be a more accurate method to achieve adequate nutrition in preterm infants according to energy and protein intakes recommended by the European Society for Paediatric Gastroenterology Hepatology and Nutrition (ESPGHAN) [25]. However, this opinion is not unanimous, as other authors found that the target HM fortification method, as compared to the standard HM fortification, did not meet the recommended protein intake [26] or result in better growth outcomes [27]. The target fortification method has also been described as inconvenient, as a result of requiring a HM analyzer and being time-consuming and labor-intensive [27].

In order to comply with the ESPGHAN enteral nutrition recommendations for preterm infants in clinical practice [25], more studies which take into account the osmolality of feeds, feeding tolerance, and risk of necrotizing enterocolitis are needed [27], in order to determine the safe maximum concentrations of multi-nutrient fortifiers and modular protein supplements in the prescribed fluid intakes.

The current literature indicates that extremely and very preterm infants at term-equivalent age have a lower fat-free mass (FFM) and a greater fat mass percentage (FM%), compared with term-equivalent-age infants [28,29]. Reference values for body composition have been described for preterm infants [30,31], but the optimal body composition evolution in those infants remains to be determined. This lack of knowledge relates not only to the difficulty in defining the optimal nutrition intervention in preterm infants, a major factor determining body composition, but also to the current unavailability of a non-invasive, accurate, and easy to perform method for measuring body composition in fragile preterm infants under intensive care, including soon after birth [32,33].

### 1.4. A Contribution to Answer Some Unsolved Questions

In a cohort study of very preterm infants fed standard fortified HM, we previously evaluated the association between energy and macronutrient intakes and both weight gain velocity and body composition [34,35]. We concluded that the actual energy, protein, and fat intakes based on measured HM composition were significantly lower than the assumed intakes based on estimates of HM composition [34]. This nutritional strategy is associated with a suboptimal weight gain velocity and low adiposity [35]. From this evidence, following the ESPGHAN enteral nutrition recommendations for preterm infants [25], a nutritional strategy towards HM fortification based on measured HM composition was recently introduced in our unit. It is considered important to assess the effects of the newly implemented nutritional strategy.

### 1.5. Objective

The aim of this study protocol is to assess whether the newly implemented target HM fortification method, based on measured HM composition, improves both the energy and macronutrient intakes and the quality of growth in very preterm infants, compared with standard HM fortification.

### 1.6. Hypotheses

In very preterm infants, the customized HM fortification based on measured HM composition is expected to improve energy and macronutrient intakes, compared with the standard HM fortification. As a consequence of better nutrition provided by the target HM fortification, the quality of growth will improve, reflected by faster growth rates while achieving an adiposity at term-equivalent age similar to that of infants born at term.

## 2. Methods

### 2.1. Study Design

This single-center, observational mixed-cohort, effectiveness study in very preterm infants (<33 weeks of gestation) will compare the energy and macronutrient intakes and the growth and body composition between infants fed either target fortified HM, based on measured HM composition (the contemporary cohort), or standard fortified HM (the historical cohort).

### 2.2. Primary Outcome

We hypothesize that the HM fortification method based on measured HM composition will result in higher energy and macronutrient intakes and faster weight gain velocity than previously observed [35]. Weight gain velocity was chosen as the primary outcome to estimate the sample size because it is considered an important measure of preterm infants’ health [10,36].

### 2.3. Secondary Outcomes

In addition, we hypothesize that in infants fed target fortified HM, adequate linear and head growth velocities will be achieved [37,38], and adiposity at term-equivalent age will be more similar to that of term-equivalent-age infants [28,29].

### 2.4. Setting, Participants, Study Periods, and Variables

The study will be conducted at the Centro Hospitalar Universitário de Lisboa Central, in the Neonatology Unit and Human Milk Bank at Maternidade Dr. Alfredo da Costa, and the Nutrition Laboratory at Hospital Dona Estefânia.

Recruitment for the contemporary cohort of infants fed target fortified HM was estimated to start in February 2020, with a scheduled recruitment period of 16 months. We planned to compare it with a historical cohort fed standard fortified HM in 2014–2015 [34,35], as stated in the ClinicalTrials.gov NCT04400396 registry. However, there was a shortage in modular protein supplement from February to July 2020, precluding the adoption of the new nutritional protocol using the target HM fortification. In this context, the standard fortified HM arm will include infants from two cohorts: the historical study period (1 February 2014 to 28 February 2015) [33,34] plus the contemporary period from 1 February to 13 July 2020, during which the standard HM fortification was used.

The recruitment for the second contemporary cohort, including infants fed target fortified HM based on measured HM composition, effectively started on 14 July 2020 (Table 1).

**Figure 1:**
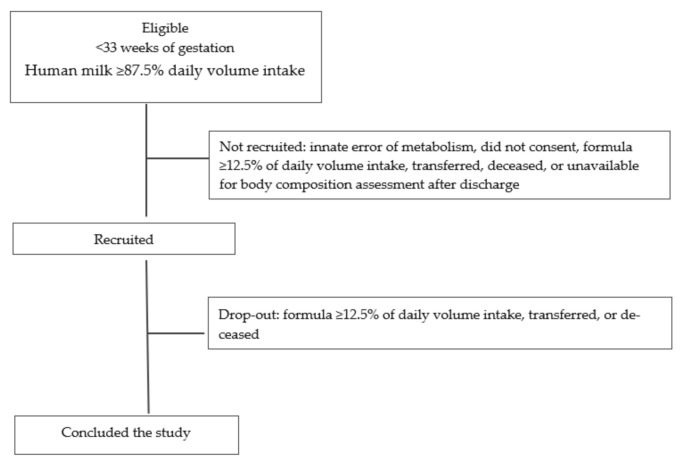
Flow diagram including the eligibility, recruitment, and drop-out criteria, used in both study arms: standard and target human milk fortification.

The eligibility criteria are those followed by the historical cohort study [34,35], i.e., consecutive cases of newborns delivered <33 weeks of gestation, admitted to the NICU of Maternidade Dr. Alfredo da Costa (inborns and outborns), singletons or twins (=2), and exclusively HM-fed or predominantly (≥87.5% volume per day) HM-fed were eligible. Infants with a diagnosis of innate error of metabolism, those fed for two or more consecutive days with formula ≥12.5% of daily volume intake, and those transferred to other hospitals, deceased, or unavailable for body composition analysis after discharge will be excluded (Figure 1).

The study sample size was calculated based on the results obtained by Macedo et al. [35] with the present historical cohort (mean 10.1, SD 3.8 g/Kg/d) and those obtained by McLeod et al. [27] on a similar sample of very preterm infants fed a target HM fortification (mean 12.1, SD 1.6 g/Kg/d). Therefore, the sample was estimated to detect a difference of 2 g/kg/day in weight gain velocity, with a significance level of 0.05 and 80% power; thus, we estimated a required sample of 68 infants: 34 infants in each cohort.

### 2.5. Nutrition Protocol

Infants will be managed according to the NICU nutrition protocol, taking into account international and national guidelines for parenteral and enteral nutrition [25,39,40,41,42,43]. Parenteral nutrition is initiated within the first 2 postnatal hours and early trophic feeding within the first 2 to 4 postnatal days using, preferentially, their mother’s own milk, and when this is insufficient, using donor’s milk. Enteral nutrition is increased as the parenteral nutrition is reduced. Nutritional schedule is as prescribed at the discretion of the attending physician. A powdered multi-component HM fortifier (Aptamil FMS; Danone GmbH, Friedrichsdorf, Germany), containing total energy 3.47 Kcal, protein 0.25 g, and carbohydrates 0.62 g per g of powder, will be started at a dose of 4.4 g/100 mL HM when the HM intake is at least 80 mL/kg/day.

For the fortification method based on measured HM, mothers’ own milk will be analyzed once a week and donor’s milk will be analyzed after the pasteurization. According to the measured HM composition, powdered modular protein (Aptamil Protein Supplement; Danone GmbH, Friedrichsdorf, Germany), containing total energy 3.38 Kcal and protein 0.821 g per g of powder, and medium-chain triglycerides (MCT OIL; Danone, GmbH, Friedrichsdorf, Germany), containing total energy 9.0 Kcal and 1.0 g *per* 1 mL, will be added to fortified HM to reach the target energy and protein daily intakes of total energy 110–135 Kcal/Kg and protein 3.5–4.0 g/Kg [25].

### 2.6. Collected Variables

#### 2.6.1. Retrieved Data

The recorded demographic variables include gestational age, sex, singleton or twin, birth weight, small-, appropriate-, or large-for-gestational age (<3rd percentile, ≥3rd percentile and ≤97th percentile, >97th percentile, respectively) [43], severity index (SNAPPE II) [44], use of prenatal corticosteroids, diagnosis of late sepsis [45], necrotizing enterocolitis (grade ≥ 3) [46], intraperiventricular hemorrhage (grade ≥ 3) [47], multiquistic periventricular leukomalacia [48], and chronic lung disease [49].

#### 2.6.2. Measurements

Analysis of HM composition. The HM will be analyzed using the Miris human milk analyzer (Miris AB, Uppsala, Sweden) [50]. To minimize daily variability in breast milk composition, mothers have been asked to add milk collected in 24 h to the same container. The composition is expressed in densities: Kcal/dL of energy and g/dL of fat, raw and true protein, carbohydrates, and ashes.

Assessment of nutrient intake. Daily intakes of energy (Kcal/kg), protein (g/kg), and fat (g/kg), and protein/energy ratio (P/E), provided by administered HM (mL/kg), non-supplemented or supplemented with multi-component HM fortifier and modular protein and fat supplements will be calculated. An Excel program designed to facilitate the calculations of the amount of modular protein and fat supplements to be added to fortified HM was developed and registered (Nona R, Cardoso M, Portuguese Directorate of Intellectual Property Services, IGAC-DSPI, no 480/2020, 26 February 2020).

Assessment of growth and body composition. During the hospital stay, the body weight is routinely measured by the attending nurses, using scales incorporated in incubators or external automatic scales. Weight gain velocity (g/kg/day) is calculated using an exponential model [51]. The same observer (MMC) measures the length and head circumference weekly, according to recommended methods [52,53], to calculate linear and head growth velocities (cm/week). At term, postmenstrual age (around 40 weeks), the fat mass (FM), FFM, %FM, percentage of FFM (%FFM), and FM index (FMI) are assessed using displacement plethysmography (Pea Pod, Cosmed, Italy), as previously described [35]. Both %FM and FMI will be used as indicators of adiposity [35].

### 2.7. Statistical Analysis

The collected data will be introduced into a Microsoft Excel database (Microsoft Corporation, 2018. Microsoft Excel, available at: https://office.microsoft.com/excel). The Epidemiology and Statistics Office of the Research Center of Centro Hospitalar Universitário Lisboa Central will perform the statistical analysis. To consider the autocorrelation structure due to the longitudinal nature of the study, generalized additive mixed-effect regression models will be used to identify the variables that explain the variability of each continuous outcome variable. Independent variables will be considered throughout the univariable analysis, and all variables with *p* < 0.25 will be selected for the multivariable models. A level of significance of α = 0.05 will be used, although *p*-values greater than 0.05 and lower than 0.1 (weak evidence of difference/association) will eventually be considered. Data will be analyzed using R (R: A Language and Environment for Statistical Computing, R Core Team, R Foundation for Statistical Computing, Vienna, Austria, 2019, http://www.R-project.org.).

### 2.8. Ethical Issues and Study Registry

The present study was approved by the institutional ethics committee (Nr 558/2018). The recruitment of participants requires the informed written consent obtained from the mothers. The study is registered at www.clinicaltrials.gov ID: NCT04400396.

## 3. Discussion

### 3.1. Strengths

The fortification of HM based on its composition is guided by direct measurements, using a validated method [54], instead of relying on estimates of HM composition reported in systematic review data [55]. In addition, the amounts of modular protein and fat supplements added to the fortified HM will be accurately calculated using an informatics tool that has been specifically developed.

Anthropometric changes will be assessed by weight, length, and head circumference growth rates, since they are more sensitive than examining the anthropometric parameters plotted on growth curves [37,38]. The weight gain velocity will be calculated by the exponential method [51], which is recommended in clinical and research settings and has been proven to provide similar results as the previously described Average2pt method [56].

The quality of growth relies on body composition assessed by air displacement plethysmography, an accurate two-compartment method validated in preterm infants [57,58], which is non-invasive, rapid to perform, and not affected by movements [33]. In addition to the %FM, adiposity will be assessed by the FMI because it is considered to be a more reliable indicator of adiposity [59], including in preterm infants [60].

### 3.2. Limitations

A randomized controlled longitudinal trial comparing methods of HM fortification would be the ideal study design to assess which method of HM fortification better optimizes energy and nutrient intakes and the quality of growth in very preterm infants. However, an interventional design has ethical implications in assigning infants to the standard HM fortification group, as an HM analyzer is available and the target HM fortification method is considered by most authors [21,22,23,24] to be the best practice in terms of complying with the high energy and protein intakes recommended by the ESPGHAN [25]. In this context, we chose an observational study design, comparing two nutritional practices sequentially adopted in our unit, i.e., the previously used standard HM fortification and the newly adopted HM fortification based on measured HM composition.

As an observational study, the prescription of the nutrition support is at discretion of the attending physicians, and we expect that they will lean towards the newly adopted target HM fortification method. Even without reaching the target energy and protein intakes recommended by the ESPGHAN [25], we expect that a HM fortification based on measured HM composition will provide better quality of growth than the standard HM fortification.

The ideal fortification method would be the target fortification guided by daily measurements of HM composition; however, this is not feasible in our setting since it would be excessively time-consuming, expensive, and labor-intensive [27]. Rochow et al. [61] assessed a cost-effective frequency for the measurement of HM composition using the target fortification method and found that measurements twice a week offer an acceptable cost benefit to enhance macronutrient intake in extremely preterm infants. We will use an alternative schedule proposed by Parat el al. [62], based on a weekly analysis, which seems to be sufficient to achieve that purpose in very preterm infants.

To assess body composition, we will use a two-compartment model that does not provide regional body composition. The ideal assessment would be longitudinal regular measurements from birth, assessed through a validated method providing whole and regional body composition, and the results would be compared with normative values obtained from “healthy” preterm infants. These standards have not been constructed yet and a non-invasive, accurate method assessing whole and regional body composition, including in fragile ventilated infants, from the first postnatal days, is still not available [32,33]. In addition, a single measurement is only scheduled after discharge, as the equipment is located in the pediatric hospital of the hospital center, not in the maternity ward where the neonatal unit is located.

A cost effectiveness analysis comparing both HM fortification strategies and the achievement of the growth targets is not planned.

## 4. Conclusions

This observational study was conceived to determine, in an effectiveness setting, the best nutrition intervention in very preterm infants, following the ESPGHAN enteral nutrition recommendations [25].

We expect to disclose whether a customized target HM fortification based on measured HM composition improves both the energy and macronutrient intakes, compared with the standard HM fortification, in very preterm infants. If a better energy and macronutrient intake is achieved, we expect to observe whether the improved nutrient intake has an effect on the quality of growth.

## Figures and Tables

**Table 1:** Study periods for the standard fortification and the target fortification arms.

Standard Fortification Arm	Target Fortification Arm
– Historical study period: February 2014 to February 2025 – 1st contemporary period: 1 February to 13 July 2020	2nd contemporary period: started on 14 July 2020

## Data Availability

The data presented in this study may be available on request from the corresponding author. According to the Institutional policy, the data are not publicly available, complying with the confidentiality for the protection of personal data.

## Abbreviations

ESPGHAN	European Society for Paediatric Gastroenterology Hepatology and Nutrition
FFM	fat-free mass
FM	fat mass
FM%	fat mass percentage
FMI	fat mass index
HM	human milk
NICU	neonatal intensive care unit
